# Supplementary material for: Use of Thrombodynamics for revealing the participation of platelet, erythrocyte, endothelial, and monocyte microparticles in coagulation activation and propagation
Source: PLoS One. 2020 May 29;15(5):e0227932. doi: 10.1371/journal.pone.0227932 (PMC7259734; doi:10.1371/journal.pone.0227932)
Supplement: S1 Movie — (PPTX) [file pone.0227932.s001.pptx]

## Slide 1
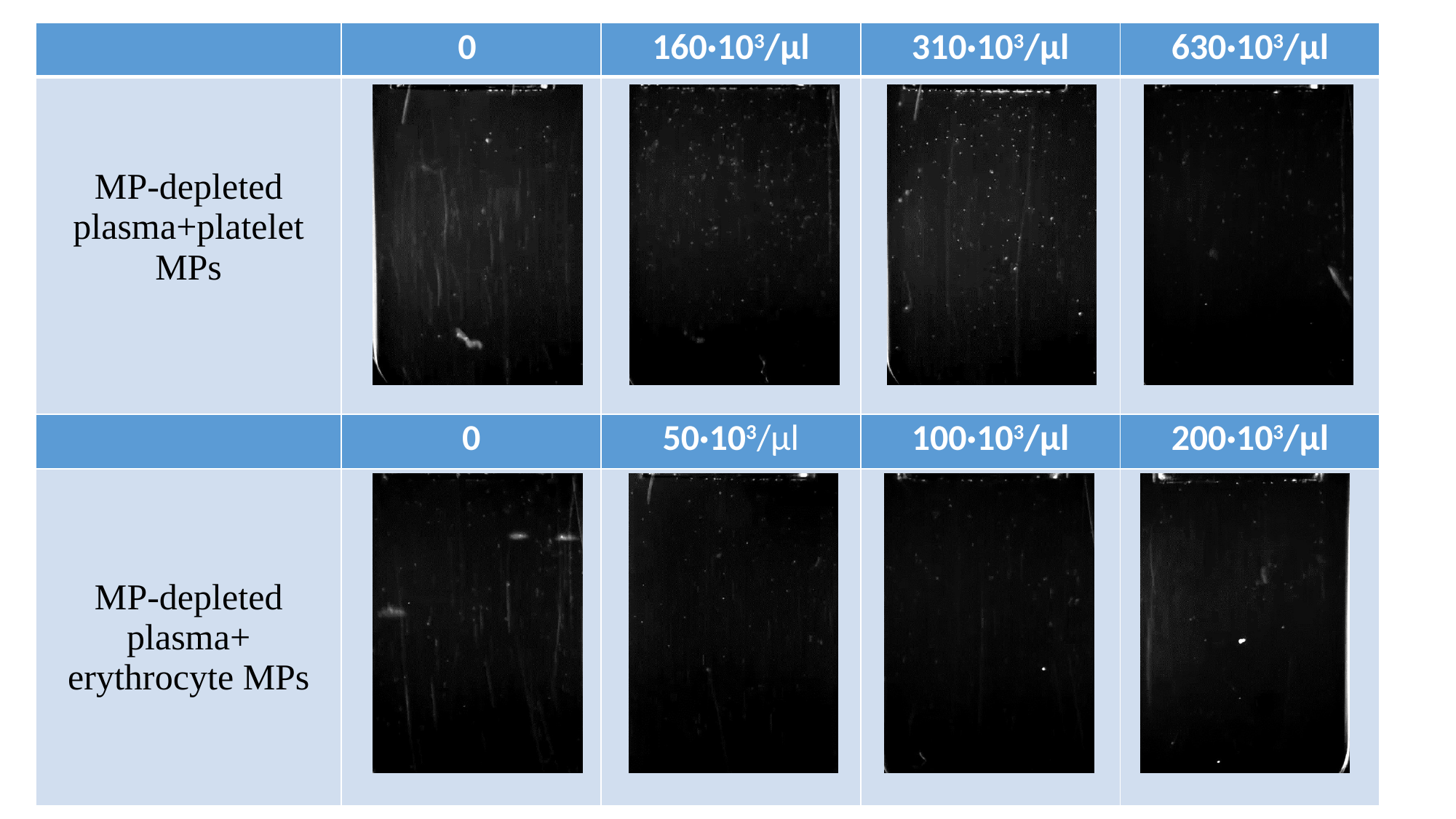

| | 0 | 160·103/µl | 310·103/µl | 630·103/µl |
| --- | --- | --- | --- | --- |
| MP-depleted plasma+platelet MPs | | | | |
| | 0 | 50·103/µl | 100·103/µl | 200·103/µl |
| MP-depleted plasma+ erythrocyte MPs | | | | |

## Slide 2
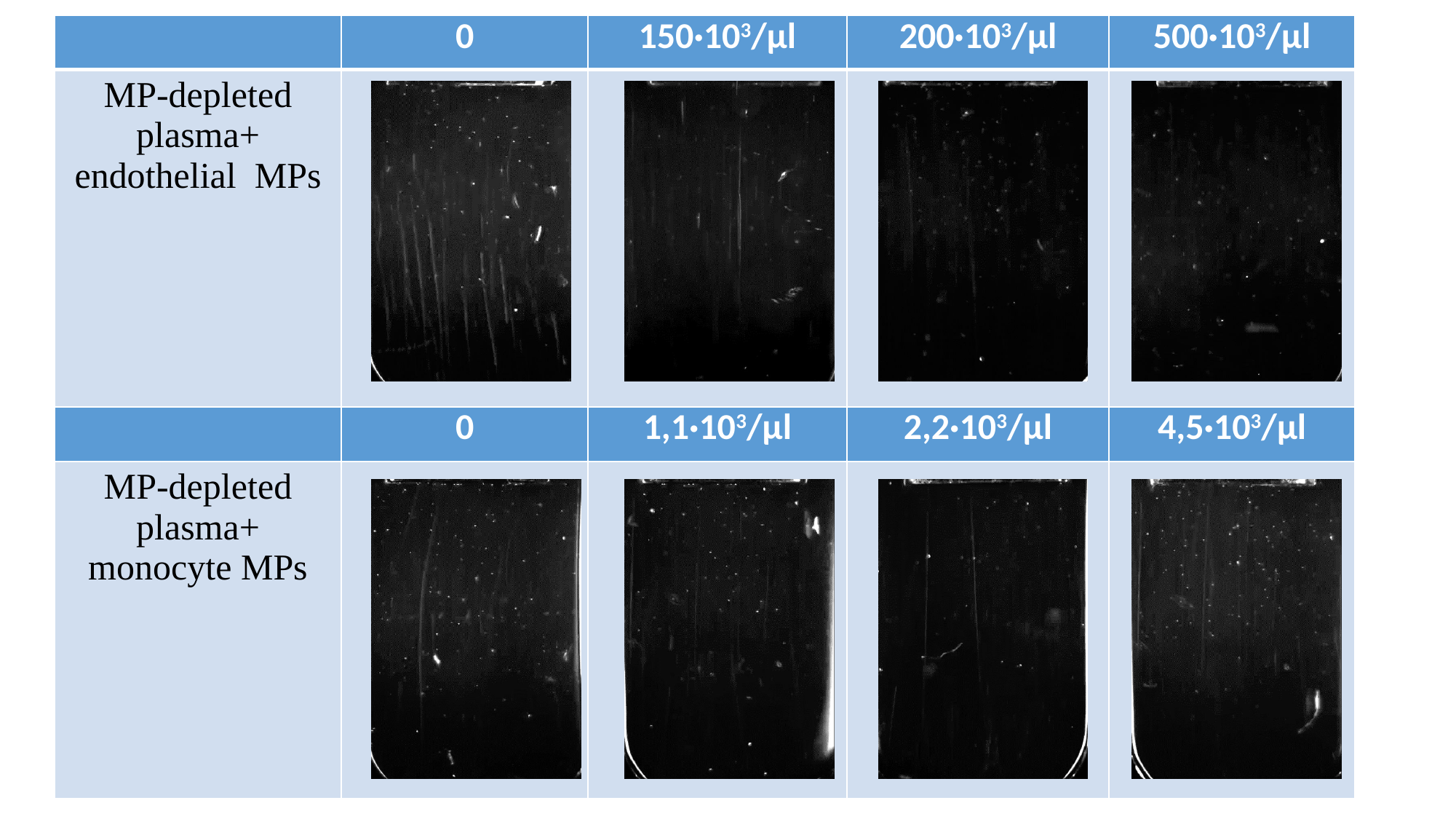

| | 0 | 150·103/µl | 200·103/µl | 500·103/µl |
| --- | --- | --- | --- | --- |
| MP-depleted plasma+ endothelial MPs | | | | |
| | 0 | 1,1·103/µl | 2,2·103/µl | 4,5·103/µl |
| MP-depleted plasma+ monocyte MPs | | | | |
