# Supplementary material for: Use of Thrombodynamics for revealing the participation of platelet, erythrocyte, endothelial, and monocyte microparticles in coagulation activation and propagation
Source: PLoS One. 2020 May 29;15(5):e0227932. doi: 10.1371/journal.pone.0227932 (PMC7259734; doi:10.1371/journal.pone.0227932)
Supplement: S1 Text — (DOCX) [file pone.0227932.s002.docx]

Kinetics of coagulation centres appearance

The time dependences of the number of clotting centres induced by MPs of different origins are represented in S1 Fig. The increment of the clot number was normalized to the fraction of free frame area so that the decrease in the rate of clotting centre appearance as a result of volume occupation by clots that appeared earlier would not distort the dependence. The scale of the ordinate axis in S1 Fig is different because the number of spontaneous clots induced by MPs of different origins within 1 hour differed by more than 100-fold. According to available data for PMPs and ErMP, the form of the clot number dependence on time is difficult to determine. For MMPs and EMPs, this dependence was exponential. It is natural to assume that the time of appearance of a spontaneous clot is determined by the local concentration of the activator in its centre. If an individual MP triggered a clot, the time of appearance of the first five centres (tN=5) should not depend on the MPs concentration. For ErMP, the assumption was confirmed (S2 Fig. B). For PMPs and EMPs, tN=5 tended to decrease with increasing MPs concentration, but the data were not sufficient for a definite answer (S2 Fig. A, C). For MMPs, tN=5 decreased inversely with MMPs concentration (S2 Fig. D). This indicates an increase in the probability of clot formation with a decrease in the distance between MMPs. The assumption about the interaction between MPs is also supported by the fact that for all types of MPs in experiments where more than 10 centres were formed within 1 hour, the increase in the number of centres was accelerated with time.

**S1 Fig. Time dependence of the number of clotting centres induced by MPs of different origins.** The number of clotting centres was recalculated to represent what it would have been if the plasma volume had not been decreased by clots that appeared earlier (N corrected). Clotting was induced in normal MP-depleted plasma by supplementation with (A) platelet MPs, (B) erythrocyte MPs, (C) endothelial MPs, and (D) monocyte MPs. Different curves correspond to different MPs samples and different concentrations. Legends identify labels of MPs samples and the concentration used in the experiment.

**S2 Fig. The dependence of the number of clotting centres formed within 60 min on concentration.** Clotting was induced in normal MP-depleted plasma by supplementation with (A) platelet MPs, (B) erythrocyte MPs, (C) endothelial MPs, and (D) monocyte MPs. Legends identify labels of MPs samples.

The dependences of the number of clotting centres formed within 60 min on concentrations are represented in S3 Fig. ErMPs in minimal concentrations that induce spontaneous clotting led to the appearance of few spontaneous clots growing at a rate near the rate of clot growth from the surface with immobilized TF (activator). On average, this rate was 48±14 µm/min. The number of spontaneous clots increased along with the ErMP concentration, but because of their fast growth, they rapidly occupied all the free chamber volume, and in our experiments, the maximal number of spontaneous clots formed within 1 hour did not exceed 17 (S3 Fig. 6). Clots induced by PMPs grew at a mean rate of 14±8 µm/min. As a result, more clots could appear at high PMPs concentrations before full coagulation (S3 Fig. A). MMPs with concentration increase did not cause coagulation at first then approximately ten clots appeared. The growth rate of these clots almost stopped within the first 10 – 15 min, and the whole plasma volume did not clot. With a further slight increase in concentration, there was a sharp switch to the formation of several hundred clots (S3 Fig. 6) growing at a relatively low but significantly nonzero rate of 9±4 μm/min on average. In this case, plasma coagulated completely and not only because of individual clots propagation in space but also because of coagulation in the whole volume. EMPs caused spontaneous coagulation patterns qualitatively similar to MMPs, but the dependence of the number of spontaneous clots on concentration was considerably smoother (S3 Fig. C), and at high EMPs concentrations, much higher clots growth rates were achieved.

**S3 Fig.** **The dependence of the number of clotting centres formed within 60 min on concentration.** Clotting was induced in normal MP-depleted plasma by supplementation with (A) platelet MPs, (B) erythrocyte MPs, (C) endothelial MPs, and (D) monocyte MPs. Legends identify labels of MPs samples.
