# Supplementary figures and images for: Use of Thrombodynamics for revealing the participation of platelet, erythrocyte, endothelial, and monocyte microparticles in coagulation activation and propagation
Source: PLoS One. 2020 May 29;15(5):e0227932. doi: 10.1371/journal.pone.0227932 (PMC7259734; doi:10.1371/journal.pone.0227932)

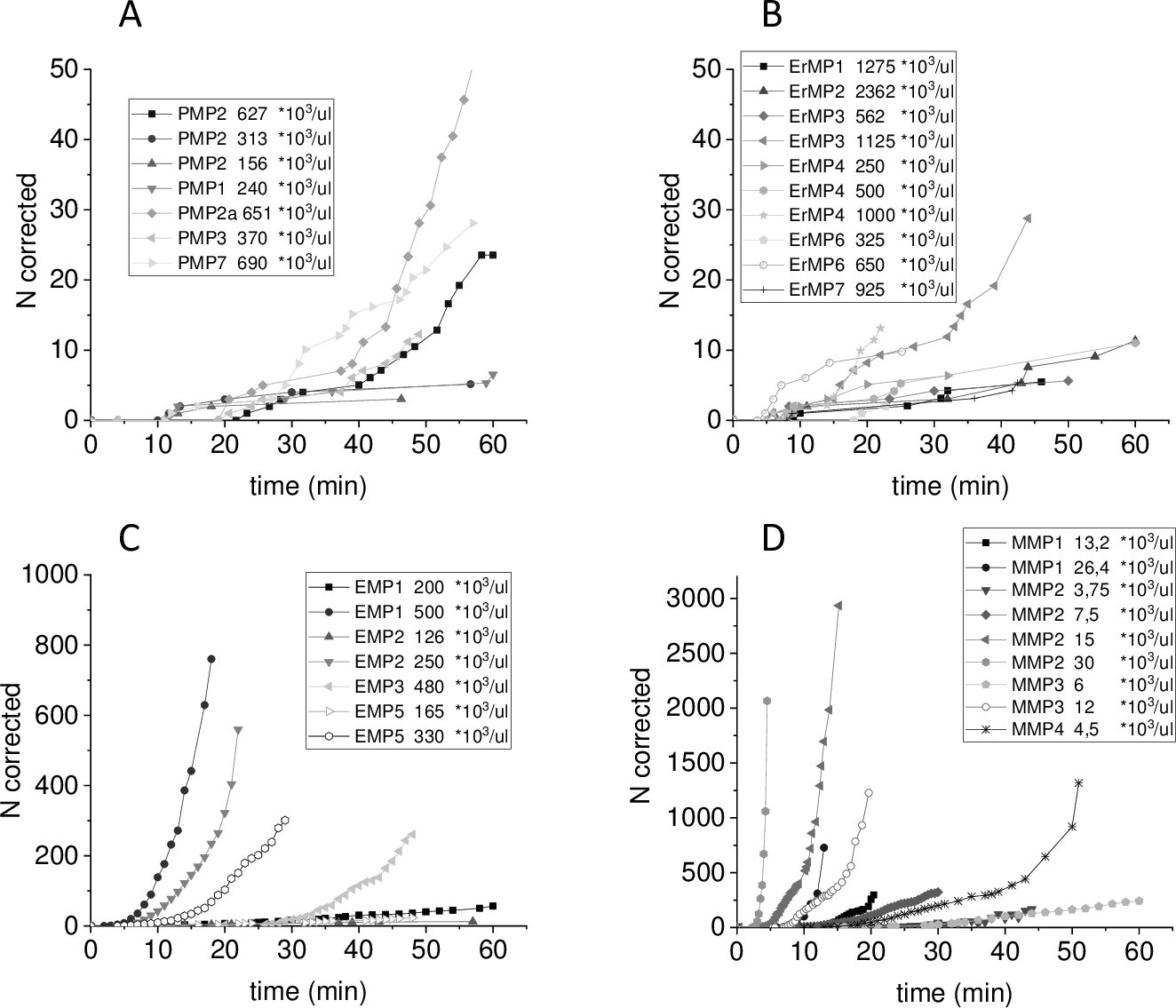

Supplement: S1 Fig — The number of clotting centres was recalculated to represent what it would have been if the plasma volume had not been decreased by clots that appeared earlier (N corrected). Clotting was induced in normal MP-depleted plasma by supplementation with (A) platelet MPs, (B) erythrocyte MPs, (C) endothelial MPs, and (D) monocyte MPs. Different curves correspond to different MPs samples and different concentrations. Legends identify labels of MPs samples and the concentration used in the experiment. (TIF) [file pone.0227932.s003.tif]

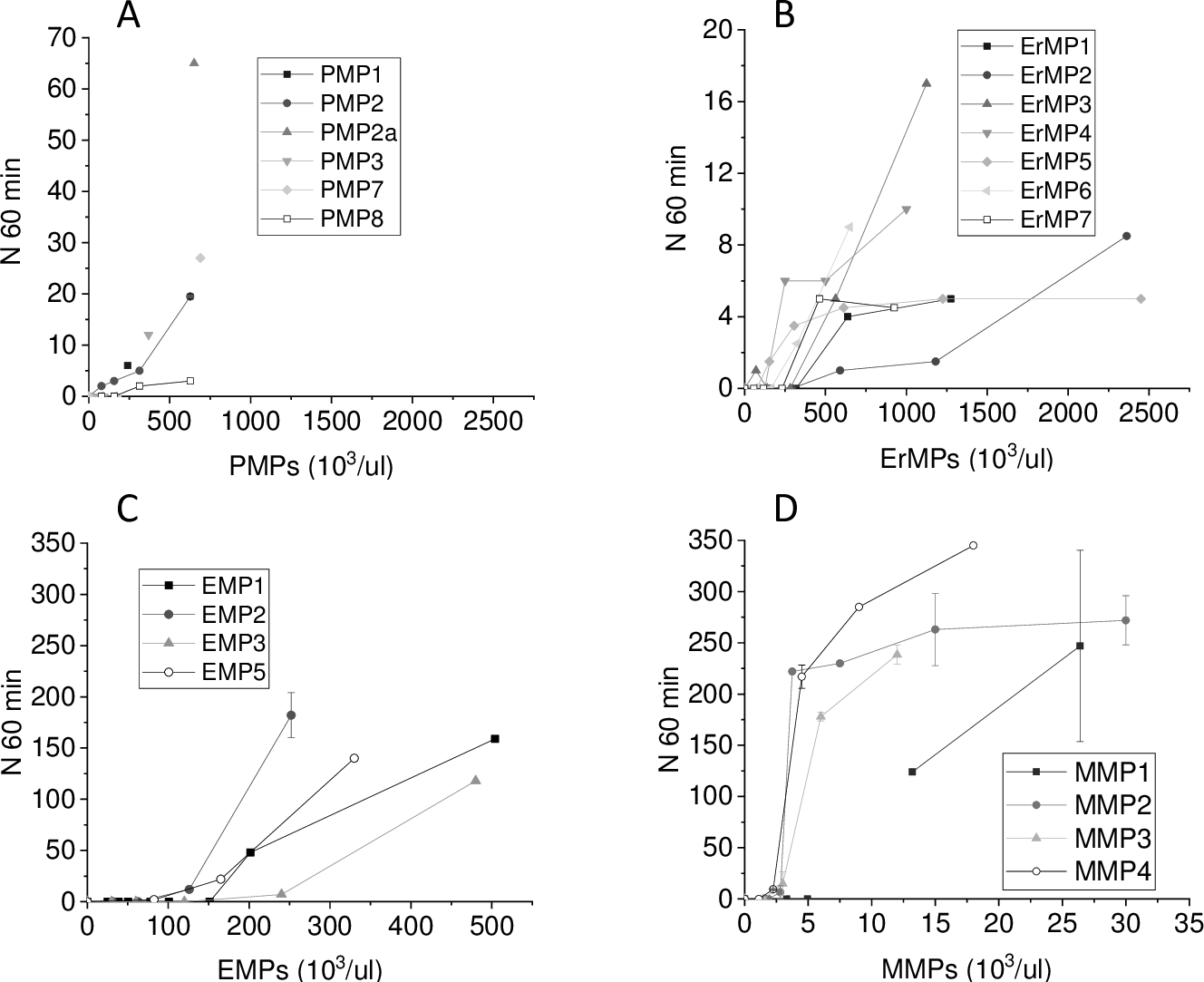

Supplement: S2 Fig — Clotting was induced in normal MP-depleted plasma by supplementation with (A) platelet MPs, (B) erythrocyte MPs, (C) endothelial MPs, and (D) monocyte MPs. Legends identify labels of MPs samples. (TIF) [file pone.0227932.s004.tif]

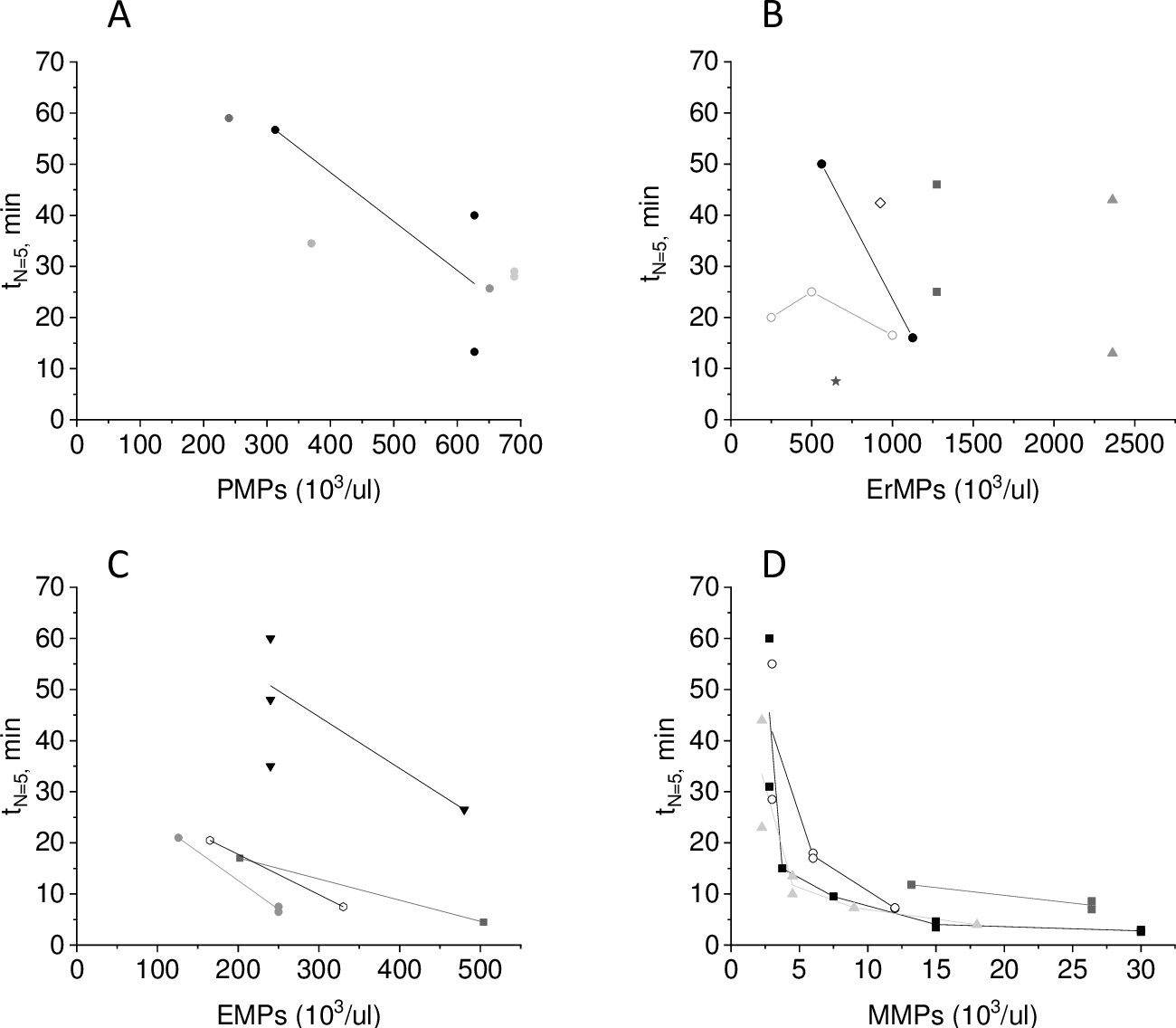

Supplement: S3 Fig — Data are represented for (A) platelet MPs, (B) erythrocyte MPs, (C) endothelial MPs, and (D) monocyte MPs. Dots correspond to individual tests, the mean values of tN = 5 at different concentrations are connected with lines, and symbols of different types and colours correspond to different MPs samples. (TIF) [file pone.0227932.s005.tif]

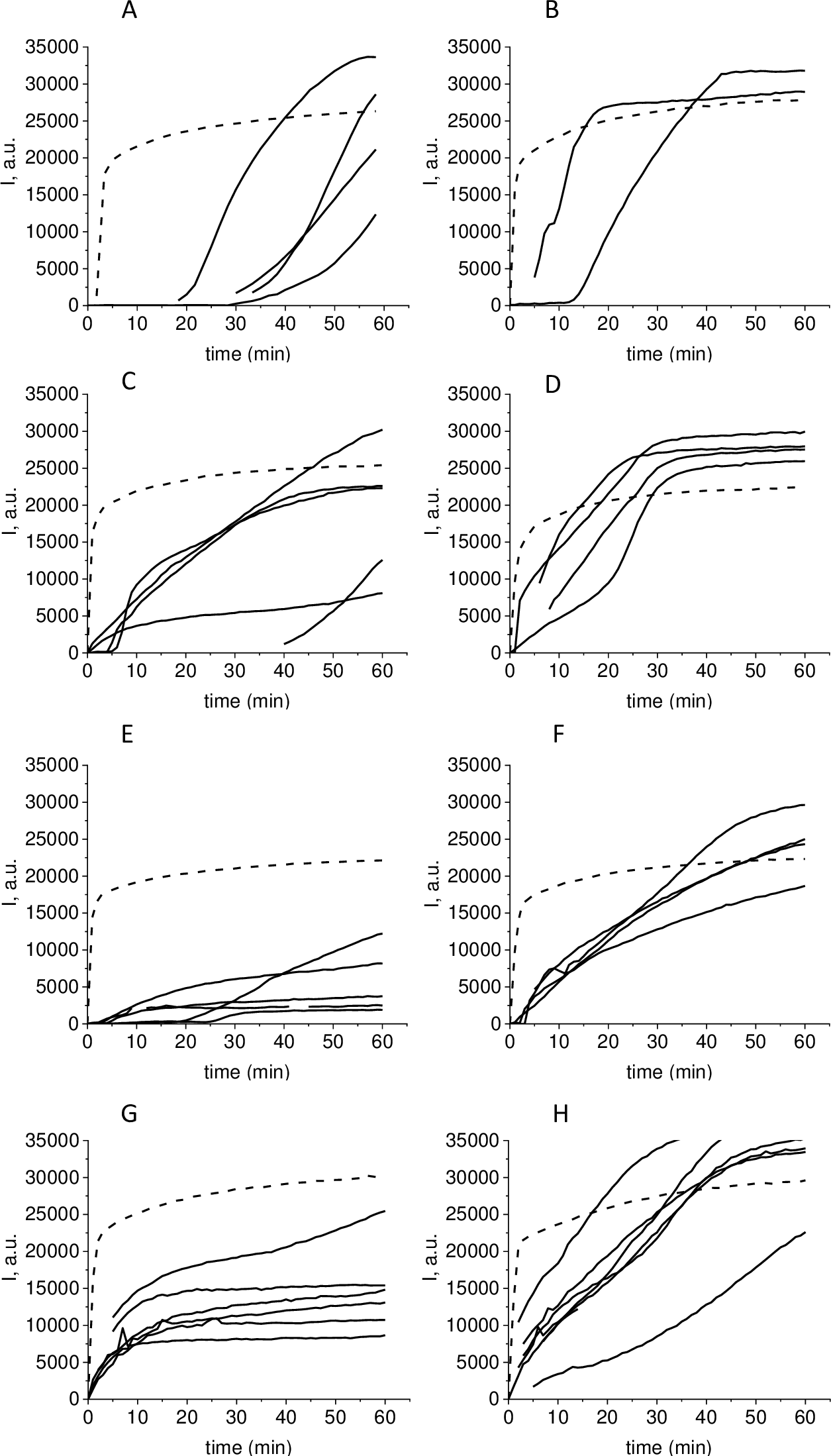

Supplement: S4 Fig — Clotting was induced in normal MP-depleted plasma by supplementation of (A) platelet MPs, (B) erythrocyte MPs, (C), (E), (G) endothelial MPs, monocyte MPs, THP MPs respectively in conditionally “low” concentrations, (D), (F), (H) endothelial MPs, monocyte MPs, THP MPs respectively in conditionally “high” concentrations. Time dependence of the light scattering intensity of clots growing from activator are drawn with dashed lines, and those of the light scattering intensity in the centre of spontaneous clots are drawn with solid lines. (TIF) [file pone.0227932.s006.tif]

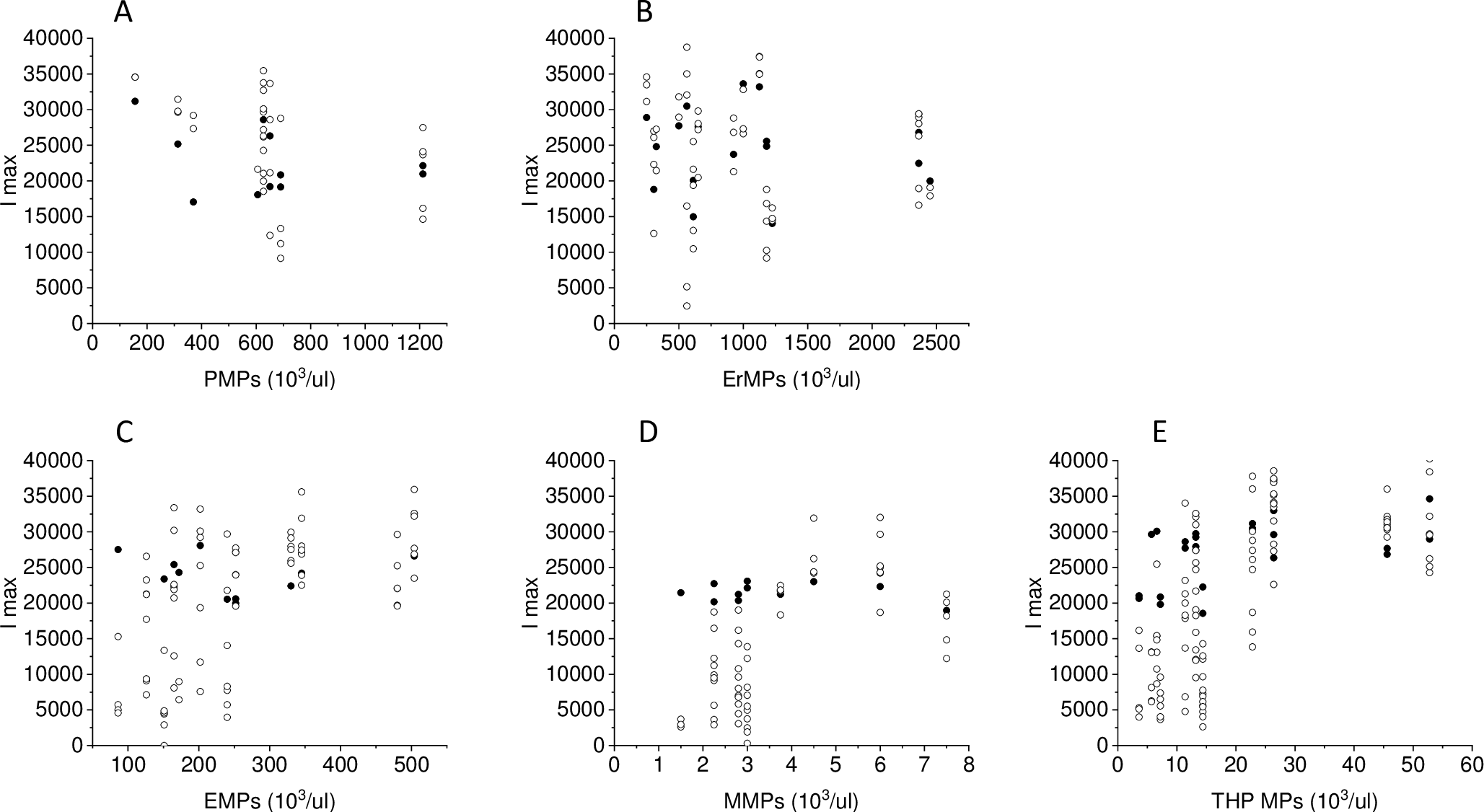

Supplement: S5 Fig — The maximal light scattering intensity dependence on the concentration of (A) platelet MPs, (B) erythrocyte MPs, (C) endothelial MPs, (D) monocyte MPs and (E) THP MPs. The maximal light scattering intensity in the centres of spontaneous clots is denoted with opened symbols, and that of clots growing from activator is denoted with filled symbols. (TIF) [file pone.0227932.s007.tif]

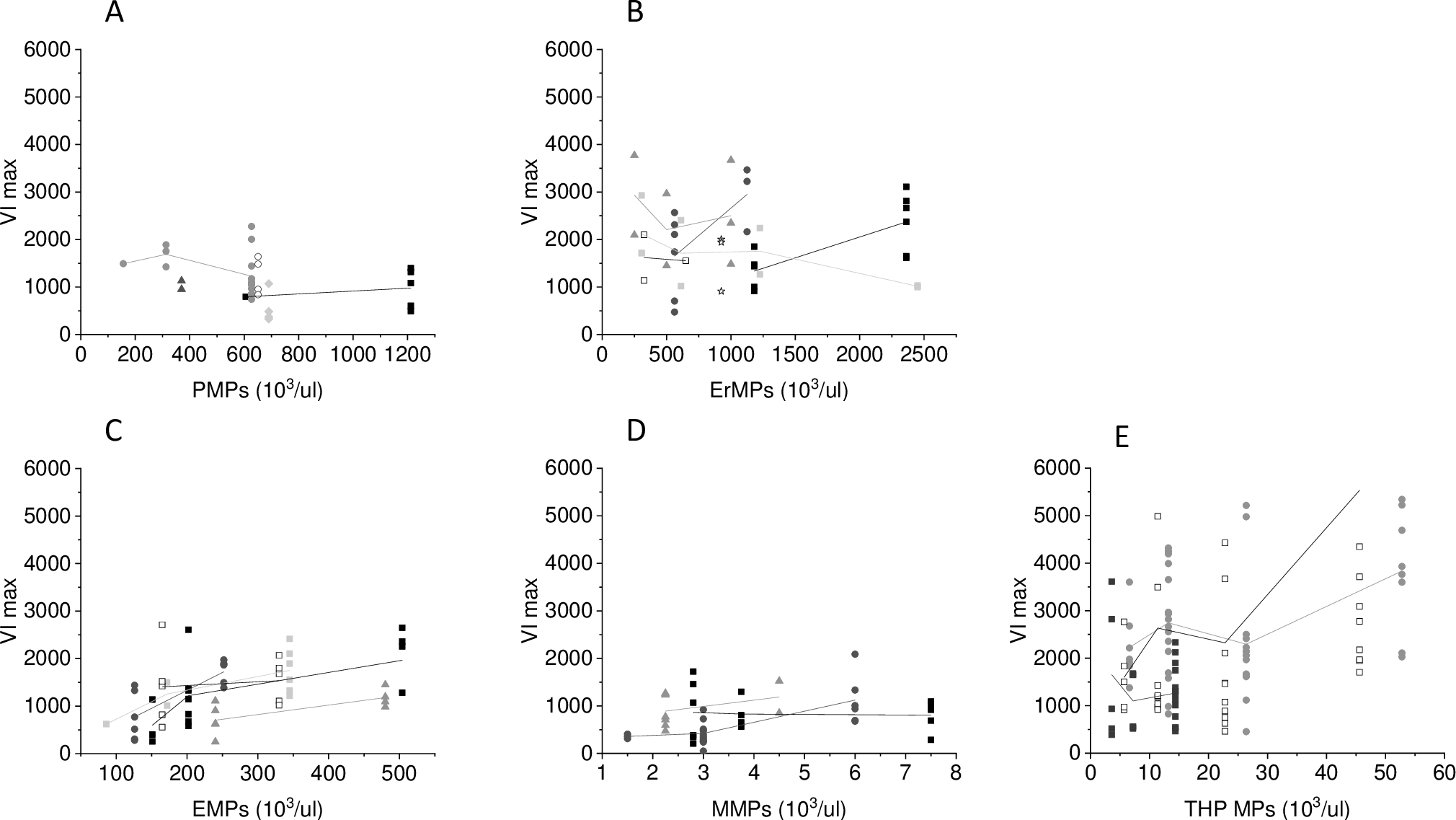

Supplement: S6 Fig — Data represent (A) platelet MPs, (B) erythrocyte MPs, (C) endothelial MPs, (D) monocyte MPs and (E) THP MPs. Dots correspond to individual tests, the mean values of VI at different concentrations are connected with lines, and symbols of different types and colours correspond to different MP samples. (TIF) [file pone.0227932.s008.tif]

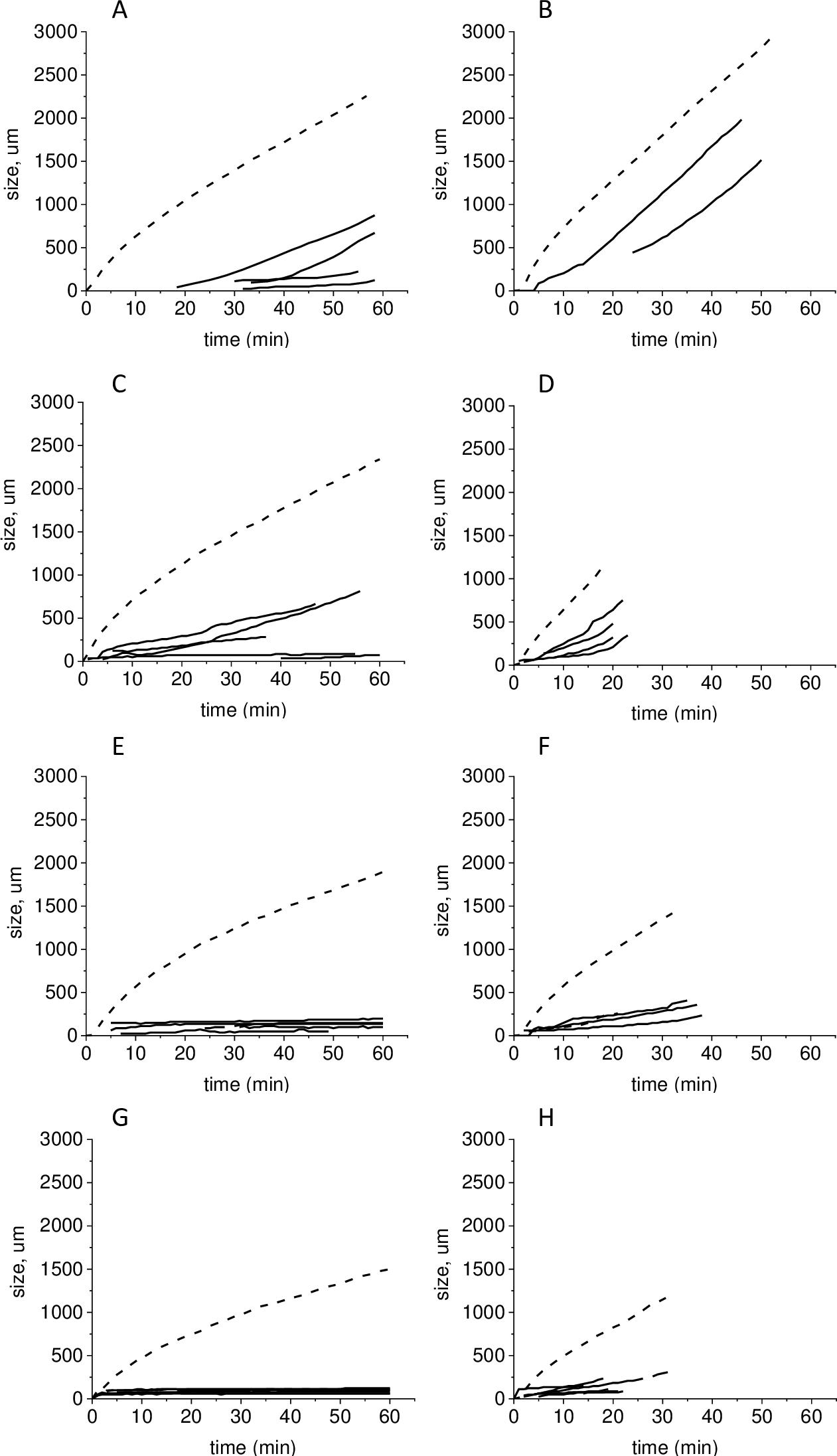

Supplement: S7 Fig — Clotting was induced in normal MP-depleted plasma by supplementation (A) platelet MPs, (B) erythrocyte MPs, (C), (E), (G) endothelial MPs, monocyte MPs, THP MPs respectively in conditionally “low” concentrations, (D), (F), (H) endothelial MPs, monocyte MPs, THP MPs respectively in conditionally “high” concentrations. Time dependences of clots growing from activator sizes are drawn with dashed lines, and those of spontaneous clot sizes are drawn with solid lines. (TIF) [file pone.0227932.s009.tif]

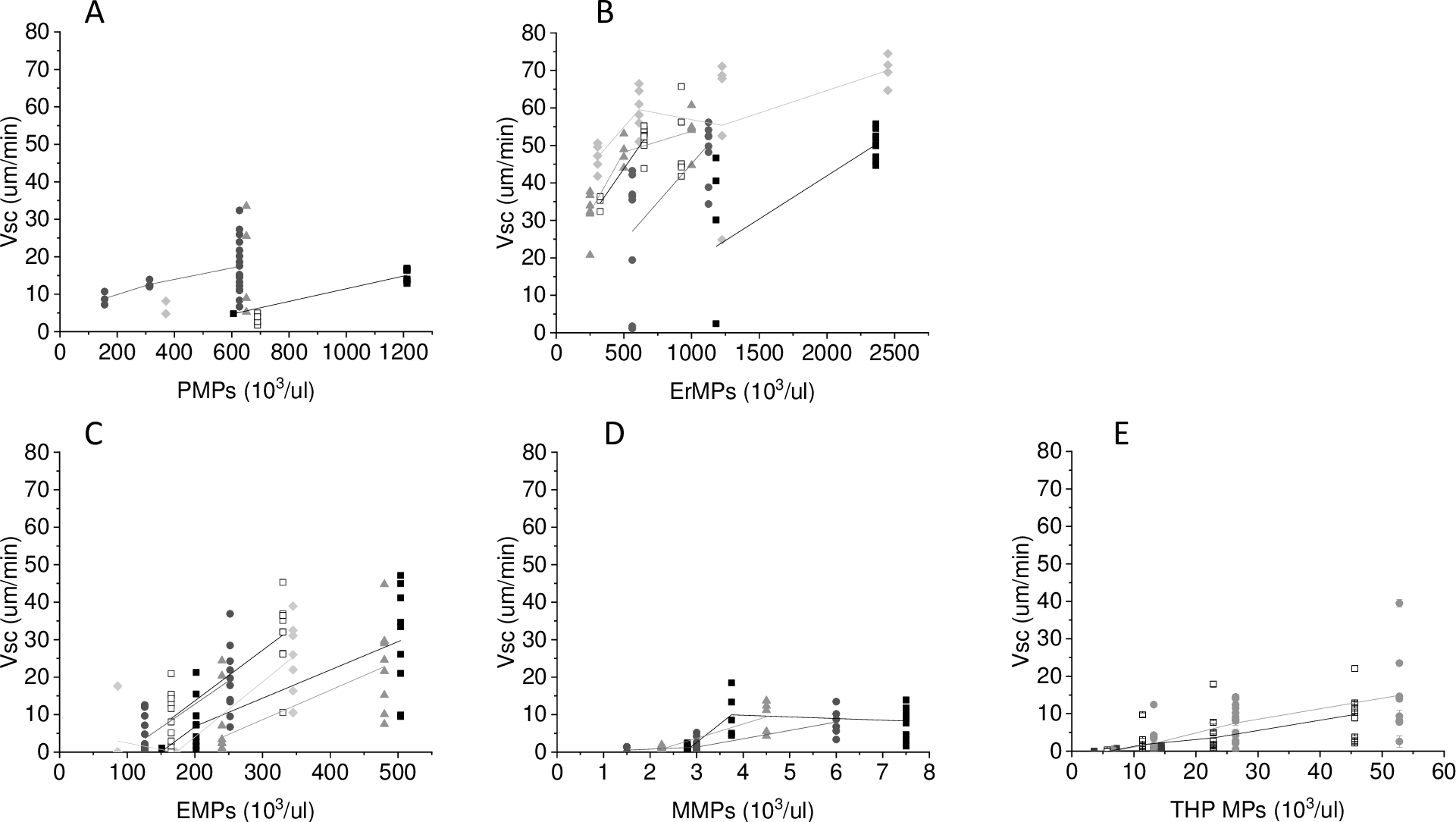

Supplement: S8 Fig — Data represent (A) platelet MPs, (B) erythrocyte MPs, (C) endothelial MPs, and (D) monocyte MPs. Dots correspond to individual tests, the mean values of VI at different concentrations are connected with lines, and symbols of different types and colours correspond to different MP samples. (TIF) [file pone.0227932.s010.tif]

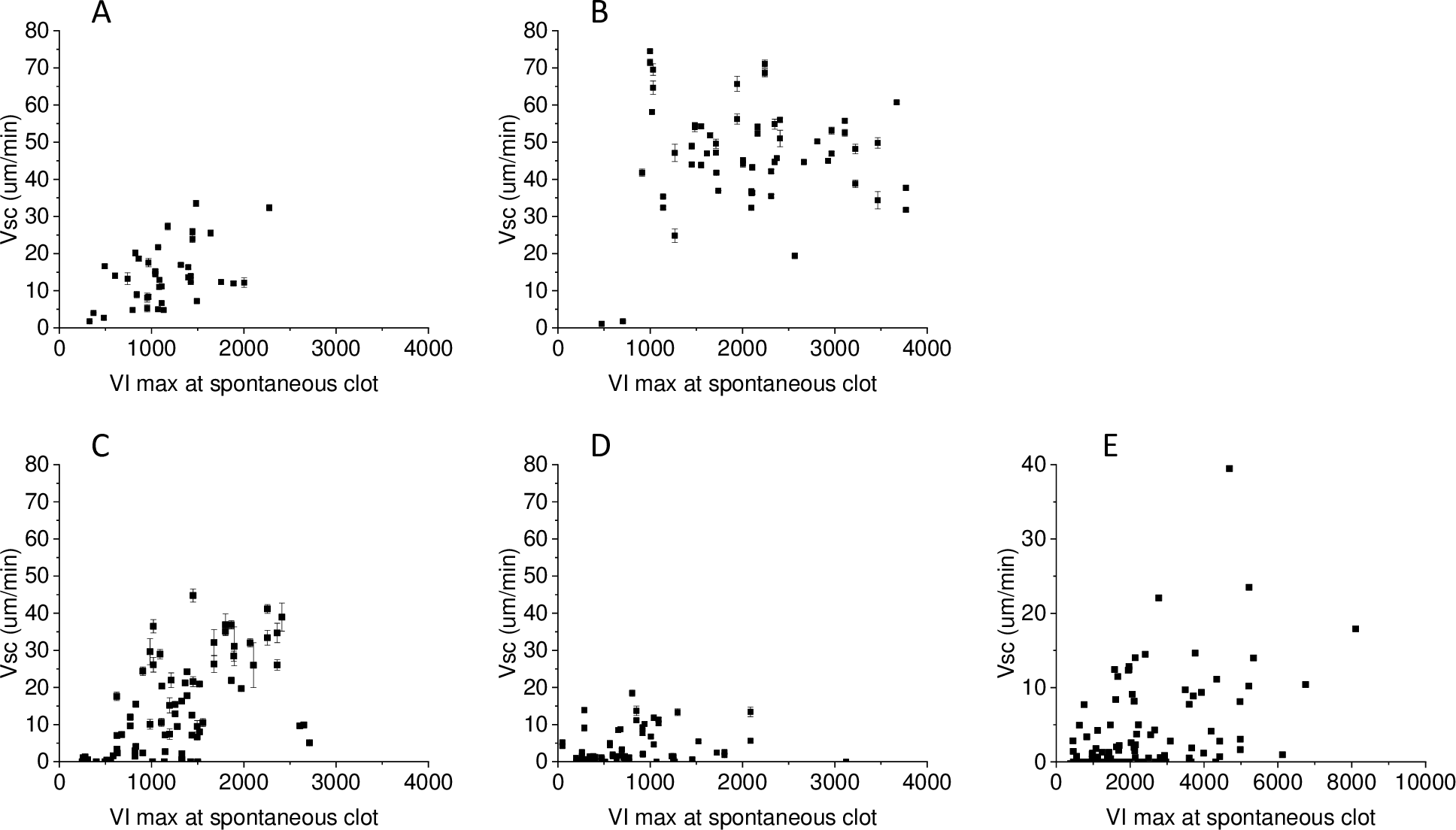

Supplement: S9 Fig — (A) Data are represented for platelet MPs, (B) erythrocyte MPs, (C) endothelial MPs, (D) monocyte MPs and (E) THP MPs. (TIF) [file pone.0227932.s011.tif]

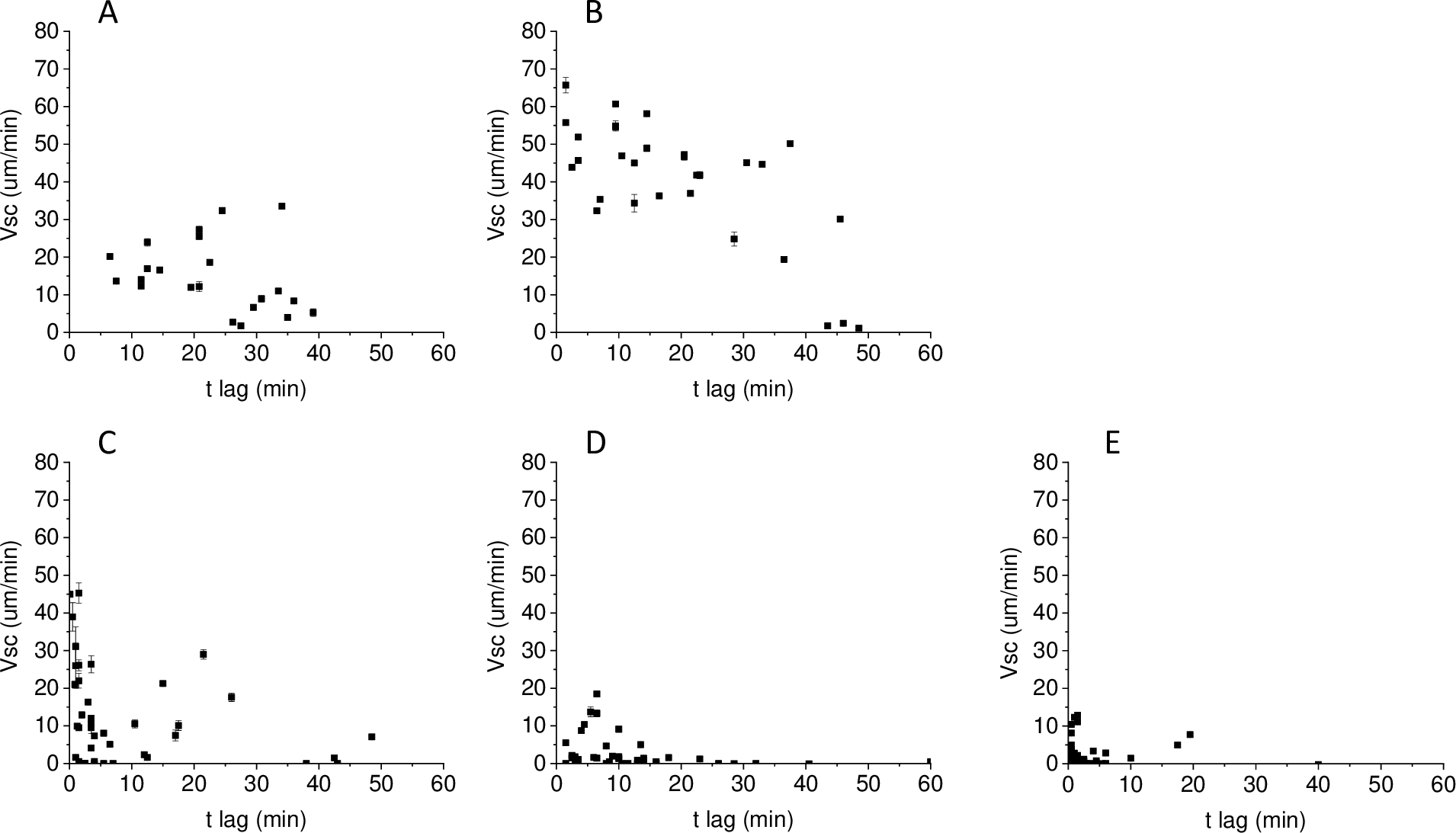

Supplement: S10 Fig — (A) Data are represented for platelet MPs, (B) erythrocyte MPs, (C) endothelial MPs, (D) monocyte MPs and (E) THP MPs. (TIF) [file pone.0227932.s012.tif]
